# Calcification of the Lateral Collateral Ligament of the Knee: A Case Series

**DOI:** 10.7759/cureus.111670

**Published:** 2026-06-28

**Authors:** Syeda Shabistan Intekhab, Alaa Al-Taie, Syed Alam, Renan Ibrahem Adam

**Affiliations:** 1 Radiology, Hamad Medical Corporation, Doha, QAT; 2 Radiology, Hamad General Hospital, Doha, QAT; 3 Medicine, Qatar University, Doha, QAT; 4 Musculoskeletal Radiology, Hamad Medical Corporation, Doha, QAT

**Keywords:** calcification, hydroxyapatite calcification, knee, knee pain, lateral collateral ligament

## Abstract

Calcification of the lateral collateral ligament (LCL) of the knee is a rare cause of lateral knee pain related to hydroxyapatite crystal deposition. We report a case series of four patients presenting with lateral knee pain. Diagnosis was established using imaging. In one case, calcification was first detected on ultrasound and subsequently confirmed on radiography and MRI. In two other cases, calcification was identified on radiography and MRI, with spontaneous radiographic resolution on follow-up in one patient. In the fourth patient, dual-energy CT was additionally performed to exclude gout. All patients were treated conservatively with nonsteroidal anti-inflammatory drugs and physiotherapy, with symptomatic improvement. Our findings suggest that plain radiography is usually sufficient to identify LCL calcification, while advanced imaging aids in excluding other diagnoses and in assessing associated soft-tissue changes. Most patients can be managed conservatively, and spontaneous resolution of calcification may be observed in some cases.

## Introduction

Calcification of the lateral collateral ligament (LCL) of the knee is a rare cause of knee pain resulting from periarticular hydroxyapatite crystal deposition. This process is well recognized in the rotator cuff tendons of the shoulder. However, involvement of the ligamentous structures of the knee is uncommon by comparison [1-3]. While calcific deposits can occur in tendons and ligaments throughout the musculoskeletal system, the LCL of the knee is a particularly rare site, with only a few cases documented in the literature [1-5].

The condition can present as acute, subacute, or chronic knee pain, most commonly localized to the lateral aspect of the joint [2,4]. It may mimic more common diagnoses such as lateral meniscal pathology, iliotibial band syndrome, LCL sprain, or crystalline arthropathy [4,6,7]. Awareness of this entity is therefore important to avoid misdiagnosis and unnecessary investigations or interventions. Most cases resolve spontaneously with conservative management, although a minority may require procedural or surgical intervention [2,3,5,6].

We present four cases of LCL calcification of the knee, each diagnosed on imaging and managed conservatively with good outcomes, and discuss this rare diagnosis in the context of the existing literature.

## Case presentation

Case 1

A 57-year-old man with no known comorbidities presented with a two-week history of left knee pain, stabbing in nature, with radiation down the lateral aspect of the lower leg. He denied fever, skin changes, or prior trauma. Pain was exacerbated by knee flexion. On examination, gait was normal. There was mild swelling of the left knee with tenderness over the lateral joint line, but no skin changes, warmth, or erythema. The active and passive range of motion of both knees was within normal limits. Valgus stress testing elicited pain; however, no ligamentous instability was observed.

Given the clinical suspicion for soft-tissue pathology, ultrasound of the knee was performed as the initial imaging modality, with a complementary plain radiograph obtained on the same day. Ultrasound revealed echogenic calcifications approximately 1.8 cm in size within the proximal course of the LCL (Figure 1). Plain radiography confirmed a calcification adjacent to the lateral femoral condyle in the region of the LCL attachment (Figure 2). MRI was subsequently obtained for further characterization and to exclude associated intra-articular pathology. It demonstrated a low-signal-intensity focus at the LCL on both T1- and T2-weighted MRI sequences, consistent with a calcific deposit, with mild surrounding soft-tissue edema. No joint effusion or other intra-articular abnormality was identified (Figure 2).

**Figure 1:**
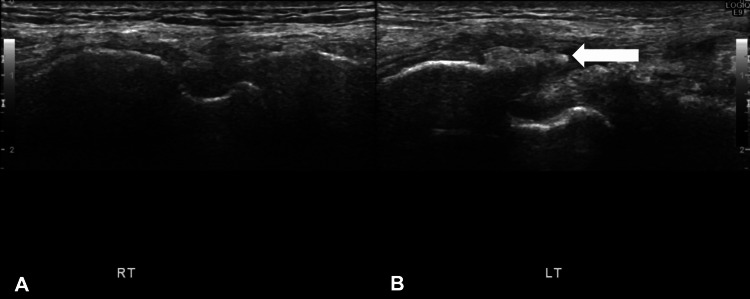
Grayscale ultrasound images of both knees. (A) Right knee shown for comparison, demonstrating no abnormality. (B) Left knee demonstrating hyperechoic calcific deposits (white arrow) with posterior acoustic shadowing adjacent to the lateral aspect of the knee joint consistent with calcification within the proximal LCL LCL: lateral collateral ligament

**Figure 2:**
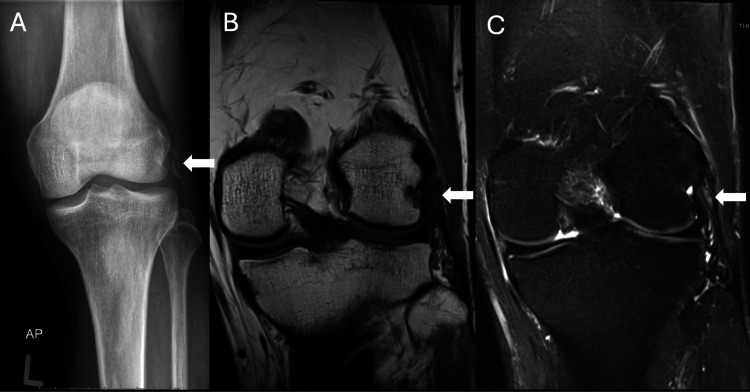
(A) AP radiograph of the left knee demonstrating a calcific opacity (white arrow) in the soft tissues adjacent to the LCL. (B) T1-weighted and (C) T2-weighted fat-suppressed coronal MRI images of the left knee demonstrating a hypointense focus (white arrow) within the LCL on both sequences consistent with calcification AP: anteroposterior, MRI: magnetic resonance imaging, LCL: lateral collateral ligament

Laboratory investigations, including white cell count, C-reactive protein (CRP), and serum uric acid, were all within normal limits. The patient was managed conservatively with nonsteroidal anti-inflammatory drugs (NSAIDs) and physiotherapy. His knee pain resolved over the next three months, with a return to normal function.

Case 2

A 52-year-old man presented with a one-week history of left lateral knee pain. On examination, there was tenderness over the lateral joint line. There was no effusion, warmth, or erythema, and range of motion and ligamentous stability were within normal limits.

Plain radiography revealed a soft-tissue calcification at the femoral insertion of the LCL on the lateral aspect of the knee (Figure 3). MRI was subsequently performed for further characterization and to exclude associated intra-articular pathology. It demonstrated a calcific deposit in the LCL with mild inflammatory changes in the adjacent soft tissue (Figure 4). No other intra-articular abnormality was identified. Laboratory investigations, including white cell count, CRP, and serum uric acid, were all within normal limits.

**Figure 3:**
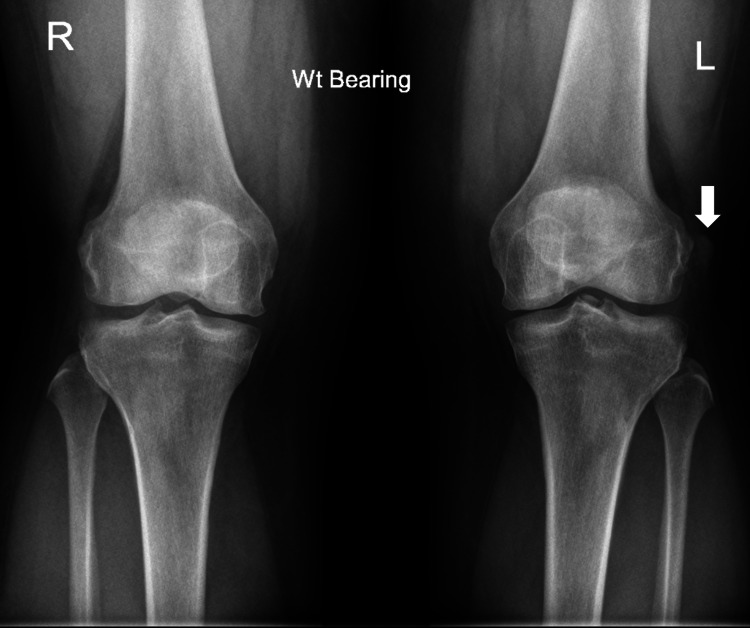
AP radiograph of both knees demonstrating a calcific opacity (white arrow) in the soft tissues adjacent to the LCL of the left knee with the right knee shown for comparison AP: anteroposterior, LCL: lateral collateral ligament

**Figure 4:**
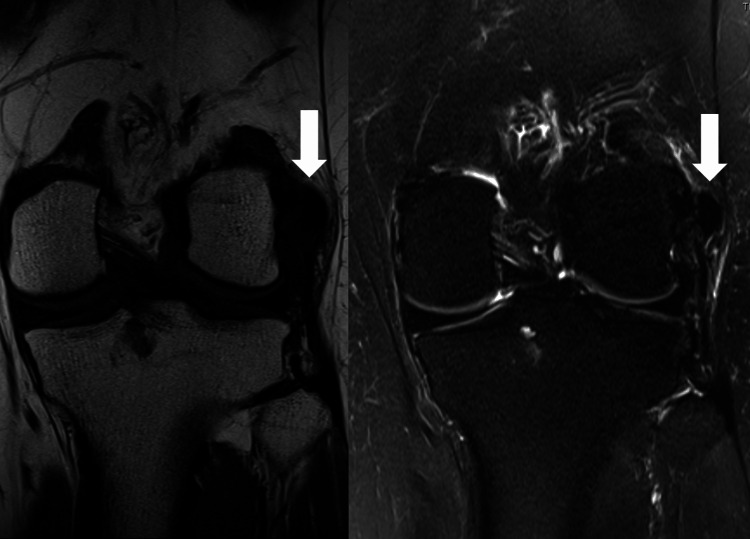
(A) T1-weighted and (B) T2-weighted fat-suppressed coronal MRI images of the left knee demonstrating a hypointense focus (white arrow) within the LCL on both sequences consistent with calcification MRI: magnetic resonance imaging, LCL: lateral collateral ligament

The patient was managed conservatively with NSAIDs and physiotherapy, with significant improvement in symptoms over approximately five weeks. At one-month follow-up, the patient reported complete relief of knee pain and had returned to normal function. A repeat plain radiograph obtained two years later demonstrated complete radiographic resolution of the previously identified LCL calcification (Figure 5). The patient reported no recurrence of symptoms at this time.

**Figure 5:**
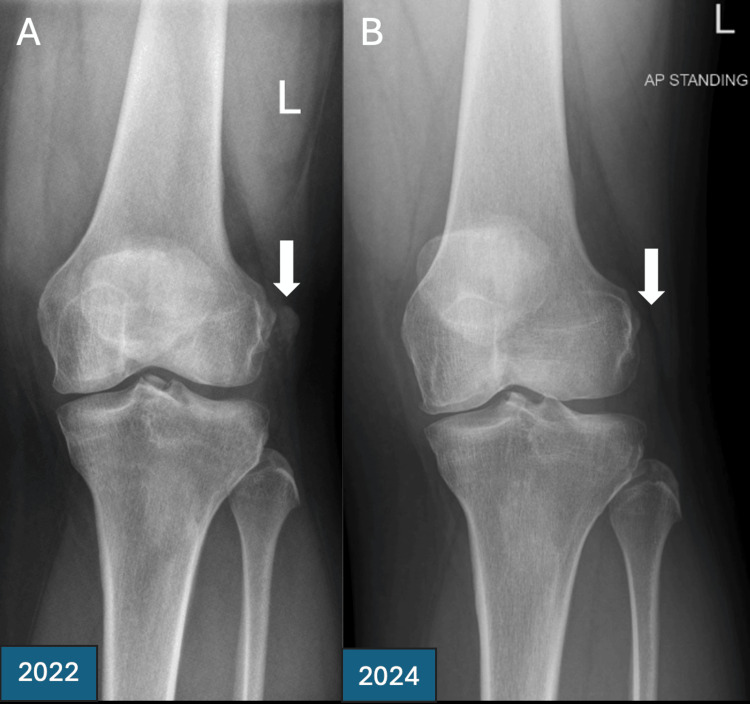
AP radiograph of the left knee. (A) Obtained in 2022, demonstrating a soft-tissue calcification (white arrow) adjacent to the lateral femoral condyle in the region of the LCL. (B) Obtained in 2024, demonstrating complete interval resolution of the previously identified calcification (white arrow) AP: anteroposterior, LCL: lateral collateral ligament

Case 3

A 38-year-old woman presented with an eight-month history of left lateral knee pain exacerbated by activity, with no history of trauma. On examination, there was tenderness along the fibular head and lateral joint line. There was no effusion, warmth, or erythema, and range of motion and ligamentous stability were within normal limits.

Plain radiography demonstrated a calcific deposit along the lateral aspect of the knee in the region of the LCL (Figure 6). MRI was subsequently performed for further characterization and to exclude associated intra-articular pathology. It identified a calcific focus in the LCL region with no other intra-articular abnormality (Figure 7). Laboratory investigations, including white cell count, CRP, and serum uric acid, were all within normal limits. The patient was managed conservatively with NSAIDs and physiotherapy, with complete resolution of symptoms at the six-month follow-up. No repeat imaging was performed, as the patient was asymptomatic at follow-up and clinical resolution was deemed sufficient.

**Figure 6:**
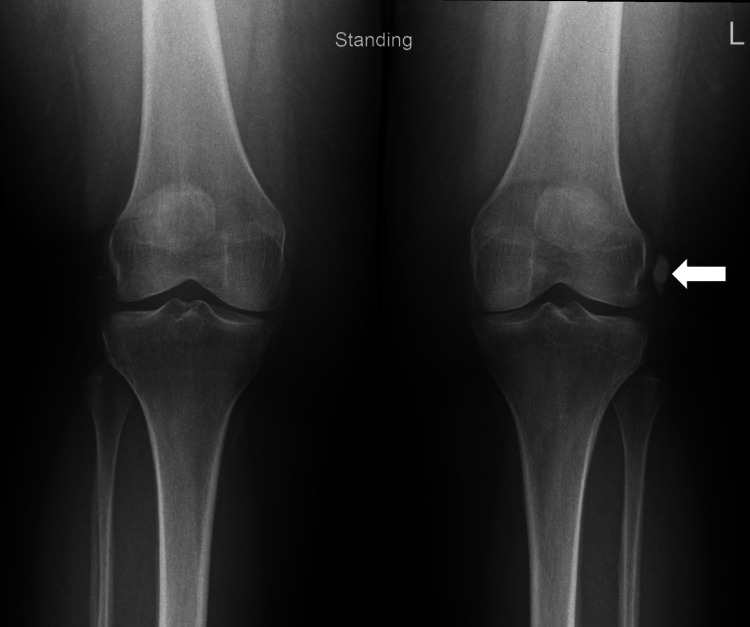
AP radiograph of both knees demonstrating a calcific opacity (white arrow) in the soft tissues adjacent to the LCL of the left knee with the right knee shown for comparison AP: anteroposterior, LCL: lateral collateral ligament

**Figure 7:**
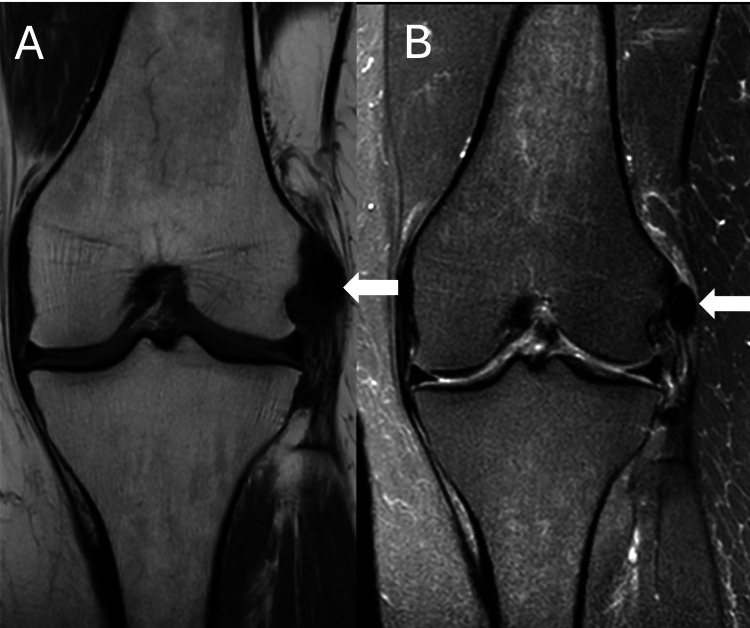
(A) T1-weighted and (B) T2-weighted fat-suppressed coronal MRI images of the left knee demonstrating a hypointense focus within the LCL (white arrow) consistent with a calcific deposit MRI: magnetic resonance imaging, LCL: lateral collateral ligament

Case 4

A 33-year-old woman presented to the emergency department with right knee pain of one year's duration, which had acutely worsened over the preceding three months to the point that she was unable to bear weight. She denied fever, constitutional symptoms, trauma, or any personal or family history of psoriasis, inflammatory bowel disease, or connective tissue disease. Examination revealed a painful, mildly swollen right knee with diffuse tenderness over the anterior joint line and suprapatellar region, without warmth or erythema. A plain radiograph demonstrated calcification in the region of the LCL (Figure 8). Point-of-care ultrasound (POCUS) confirmed a right knee effusion, which was aspirated (2 mL) and injected with triamcinolone and lidocaine, resulting in significant clinical improvement and restoration of ambulation. Aspiration fluid culture was negative, excluding septic arthritis.

**Figure 8:**
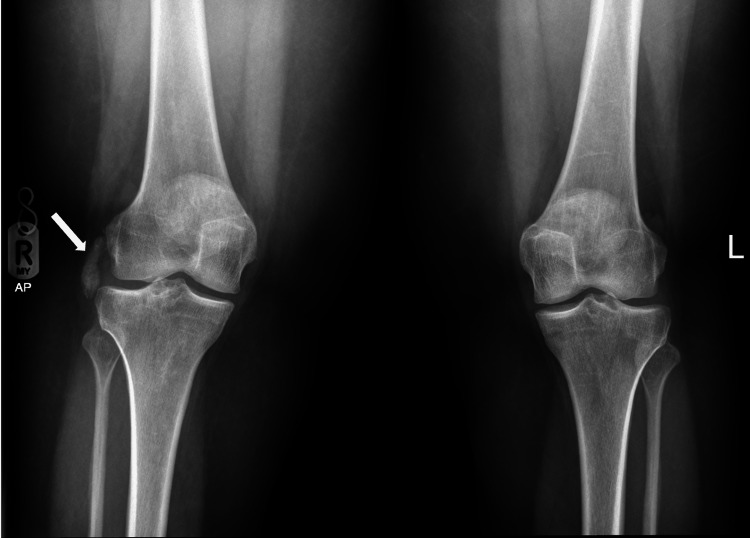
Bilateral knee radiograph demonstrating calcification (white arrow) in the region of the LCL of the right knee LCL: lateral collateral ligament

Given the diagnostic uncertainty surrounding her chronic knee pain, she was referred to a rheumatologist. MRI of the right knee demonstrated periarticular calcification extending along and deep to the LCL. Additional findings included a small, loose body anterior to the anterior cruciate ligament, chondromalacia of the patella, and extensive cystic changes of the proximal lateral tibial plateau (Figure 9). On review with the rheumatologist, she reported morning stiffness, worsening right knee pain and swelling, right shoulder pain of six months, and mild polyarthralgia without swelling in other joints.

**Figure 9:**
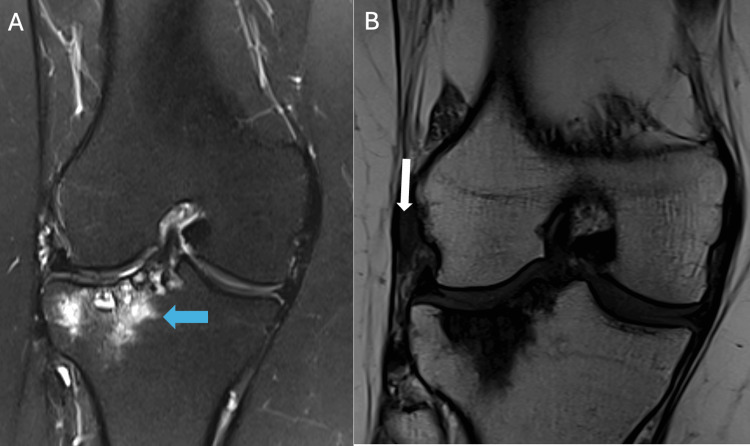
Coronal MRI images of the right knee. (A) T2-weighted fat-suppressed sequence demonstrating bone marrow edema and cystic changes in the lateral tibial plateau (blue arrow). (B) T1-weighted sequence demonstrating a hypointense focus within the LCL consistent with calcification (white arrow) MRI: magnetic resonance imaging, LCL: lateral collateral ligament

Inflammatory markers and uric acid were within normal limits. Serological investigations, including rheumatoid factor, anti-cyclic citrullinated peptide, antinuclear antibody, and human leukocyte antigen B27, were all negative. The patient was provisionally diagnosed with chronic right knee monoarthritis and possible undifferentiated inflammatory arthritis. She was started on full-dose NSAIDs. The patient was symptom-free at one-month follow-up.

Approximately one year later, the patient re-presented with a few weeks' history of recurrent right knee pain similar in character to her original presentation. Repeat investigations, including inflammatory markers and uric acid, were within normal limits. Dual-energy CT (DECT) of the knee was performed and did not demonstrate monosodium urate crystal deposition, effectively excluding gout (Figure 10). POCUS demonstrated synovial hypertrophy without significant effusion, and an intra-articular methylprednisolone 80 mg injection was administered under ultrasound guidance, resulting in symptomatic improvement. A follow-up was planned at two months to assess the response.

**Figure 10:**
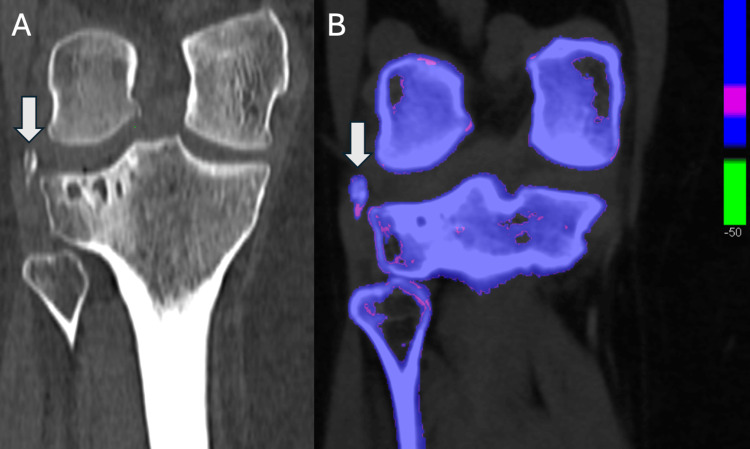
DECT of the right knee. (A) Conventional CT image in the coronal plane demonstrating cystic changes in the lateral tibial plateau and calcification within the LCL (white arrow). (B) Color-coded post-processed DECT image demonstrating calcium deposits within the LCL with no monosodium urate crystal deposition identified (white arrow) DECT: dual-energy computed tomography, CT: computed tomography, LCL: lateral collateral ligament

## Discussion

Calcification of the LCL is a rare cause of knee pain, which may present as acute or subacute pain, predominantly in the lateral aspect of the knee [2,4]. The pathogenesis of this condition is not clearly understood [2,3]. It is thought to reflect underlying hydroxyapatite crystal deposition, analogous to that seen in calcific periarthritis at other sites [2,3,4]. Symptoms are believed to arise when the calcific deposit ruptures and dissolves into the surrounding structures, triggering a local inflammatory response, which may explain why some patients remain asymptomatic [2,3]. In our series, all patients were middle-aged to older adults with no acute trauma, and none had a clearly documented precipitating factor, which is consistent with the idiopathic nature of hydroxyapatite deposition disease [2,4].

Recognizing this condition is important as it can mimic other causes of knee pain. It can be mistaken for lateral meniscal tears, LCL sprains, osteoarthritis, or even crystalline arthropathies like gout or pseudogout if calcification is noted but not correctly attributed [6,7]. Therefore, imaging plays a key role in diagnosis. Plain radiography can depict soft-tissue calcifications, though smaller calcific deposits may be missed if not carefully looked for [2,4]. In all four of our cases, calcifications were easily identified on plain radiographs. In the first case, the echogenic calcific deposits were also seen on ultrasound. Ultrasound may therefore be useful as an initial diagnostic tool, particularly when septic arthritis is a concern. In Case 4, POCUS was used therapeutically to identify and aspirate a knee effusion and subsequently to guide an intra-articular corticosteroid injection, demonstrating its utility as a real-time procedural tool in this condition.

MRI provides more detailed soft-tissue characterization and plays an important role in the exclusion of associated intra-articular pathology. Anderson et al. were among the first to describe the MRI appearances of this condition, noting that LCL calcification may appear aggressive and mimic other knee abnormalities [4]. This highlights the importance of correlating MRI findings with plain radiography and clinical context. Furthermore, Case 4 is notable for its atypical MRI appearance, with periarticular calcification extending along and deep to the LCL, accompanied by extensive cystic changes of the proximal lateral tibial plateau. This demonstrates that MRI appearances can be complex and potentially misleading if not interpreted in the appropriate clinical setting. This constellation of findings underscores that LCL calcification may not occur in isolation, and MRI remains indispensable for characterizing the full extent of periarticular and intra-articular involvement [8]. In all four of our cases, MRI provided valuable diagnostic information beyond what plain radiography alone could offer.

Management of LCL calcification is generally conservative, particularly in patients with manageable symptoms and no significant functional impairment. The majority of cases reported in the literature respond well to nonoperative treatment, with symptomatic improvement typically occurring over time and with calcific deposits often undergoing spontaneous resorption [2-5,8,9]. In our second case, radiographic evidence of complete calcification resolution was obtained within a few months of conservative therapy, highlighting that spontaneous resolution can indeed occur. All patients in our series experienced substantial pain relief with NSAIDs, rest, and physiotherapy. In Case 4, the patient presented with a significant acute exacerbation. A knee effusion was confirmed on POCUS, aspirated, and injected with an intra-articular corticosteroid, which provided rapid symptomatic relief and restored ambulation. On subsequent follow-up, when synovial hypertrophy persisted without a significant aspiratable effusion, ultrasound-guided intra-articular methylprednisolone injection again provided benefit. This suggests that in cases with an inflammatory component or acute flare, image-guided aspiration and injection represent a valuable adjunct to standard conservative measures. Case 4 also experienced a symptomatic recurrence approximately one year after initial treatment, which is a recognized pattern in hydroxyapatite deposition disease [10]. DECT at recurrence excluded gout, and repeat image-guided intra-articular injection provided symptomatic benefit. Case 4 illustrates the diagnostic complexity that can arise when LCL calcification coexists with features mimicking inflammatory arthritis. A thorough serological workup was necessary to exclude primary inflammatory and crystal-induced arthropathies. Given the favorable outcomes observed across all four cases, conservative management therefore remains the recommended first-line approach. A few cases have also been reported in which arthroscopically or fluoroscopically guided procedures were performed when conservative treatment failed to provide adequate symptom relief [1,6,11,12]. This series demonstrates that LCL calcification of the knee encompasses a broader clinical spectrum than previously described, and a multimodal approach to diagnosis and management is crucial.

This series has several limitations. The small number of cases limits generalizability. The two-year follow-up interval in Case 2 precludes precise timing of spontaneous radiographic resolution. Additionally, histopathological confirmation of hydroxyapatite crystal deposition was not obtained in any case.

## Conclusions

LCL calcification of the knee is a rare and often overlooked cause of lateral knee pain. This case series highlights the importance of considering LCL calcification in the differential diagnosis of lateral knee pain, particularly when imaging reveals periarticular calcific deposits adjacent to the lateral joint line. All four patients in our series responded to conservative measures. Cases 1 through 3 were managed with NSAIDs and physiotherapy, while Case 4 additionally benefited from POCUS-guided aspiration and intra-articular corticosteroid injection. The majority of cases achieve good outcomes without surgical intervention. Awareness of this entity can help avoid misdiagnosis and unnecessary invasive procedures.
